# Association of Eicosapentaenoic and Docosahexaenoic Acid Intake with Low Birth Weight in the Second Trimester: The Japan Pregnancy Eating and Activity Cohort Study

**DOI:** 10.3390/nu15224831

**Published:** 2023-11-18

**Authors:** Momoka Yoshimura, Megumi Fujita, Ai Shibata, Riko Ohori, Satoko Aoyama, Kaori Yonezawa, Yoko Sato, Satoshi Sasaki, Masayo Matsuzaki, Yoshiko Suetsugu, Megumi Haruna

**Affiliations:** 1 Department of Clinical Nursing, Graduate School of Medical Science, Yamagata University, Yamagata 990-9585, Japan; momo.r446@gmail.com (M.Y.); love.tm.iwtbad.06@gmail.com (A.S.); 2 Department of Midwifery and Women’s Health, Division of Health Sciences and Nursing, Graduate School of Medicine, The University of Tokyo, Tokyo 113-0033, Japan; rohori@g.ecc.u-tokyo.ac.jp (R.O.); aoyama-satoko125@g.ecc.u-tokyo.ac.jp (S.A.); kaoriyone@m.u-tokyo.ac.jp (K.Y.);; 3 Global Nursing Research Center, Graduate School of Medicine, The University of Tokyo, Tokyo 113-0033, Japan; 4 Department of Health Sciences, Graduate School of Medical Sciences, Kyushu University, Fukuoka 812-8582, Japan; satou.youko.350@m.kyushu-u.ac.jp (Y.S.); suetsugu.yoshiko.742@m.kyushu-u.ac.jp (Y.S.); 5 Department of Social and Preventive Epidemiology, School of Public Health, The University of Tokyo, Tokyo 113-0033, Japan; stssasak@m.u-tokyo.ac.jp; 6 Department of Reproductive Health Nursing, Graduate School of Health Care Sciences, Tokyo Medical and Dental University, Tokyo 113-8519, Japan; matsu3@sahs.med.osaka-u.ac.jp; 7 Department of Children and Women’s Health, Division of Health Sciences, Graduate School of Medicine, Osaka University, Osaka 565-0871, Japan

**Keywords:** docosahexaenoic acid, eicosapentaenoic acid, J-PEACH study, low birth weight, pregnancy

## Abstract

This study examined the association of eicosapentaenoic acid (EPA) and docosahexaenoic acid (DHA) intake during the second trimester with low birth weight (LBW) in pregnant Japanese women and was conducted in conjunction with the Japan Pregnancy Eating and Activity Cohort (J-PEACH) study. The study included 504 pregnant women from four Japanese sites. During the second trimester (14–27 weeks), the participants filled out a self-administered questionnaire assessing the frequency of DHA and EPA supplement intake in the past month, as well as a brief-type self-administered diet history questionnaire (BDHQ). The analysis involved data from two time points: responses to the BDHQ and infant data at birth. In total, 471 and 33 participants were classified into the normal birth weight and LBW groups, respectively. The participants were divided into high-, medium-, and low-intake groups based on their total dietary and EPA and DHA supplementary intakes. The Cochran–Armitage trend test was used to analyze the data; the prevalence of LBW was higher in the low-intake group (*p* = 0.04). There was no significant sex-based trend (*p* = 0.27 and *p* = 0.35). In Japanese women, low dietary and supplementary EPA and DHA intake until the second trimester were risk factors for LBW.

## 1. Introduction

The percentage of low birth weight (LBW) babies (weighing < 2500 g) in Japan increased from 6.3% in 1990 to 9.4% in 2019 [1] and is higher compared with that in other developed countries [2]. The short-term outcomes of LBW include higher mortality and morbidity, and the long-term outcomes include a higher incidence of lifestyle-related diseases such as impaired glucose tolerance [3,4]. In addition, it is an important determinant of the future health and development of these children.

Previous studies have reported smoking, low income, and related disorders such as gestational hypertension and chromosomal abnormalities as risk factors for LBW [5]. Additionally, inadequate maternal nutrition during pregnancy is considered a cause of LBW in several developing countries [6]. Particularly, deficiencies of eicosapentaenoic acid (EPA) and docosahexaenoic acid (DHA) in the diet have been reported to increase LBW incidence in developing countries [7,8].

EPA and DHA are *n*-3 unsaturated fatty acids (*n*-3 fatty acids) that play an important role in various biological functions; e.g., DHA is the prominent phospholipid fatty acid in the cerebral cortex and the retina [9]. *n*-3 fatty acids exert anti-inflammatory effects and cannot be synthesized in the body. It has been reported that their deficiency during the perinatal period causes inflammation, which can result in preterm delivery and gestational hypertension [10]. Therefore, consuming *n*-3 fatty acids, particularly EPA and DHA, during pregnancy is highly essential. Fish are an excellent source of EPA and DHA, but owing to the recent westernization of the Japanese diet and the rising awareness about the presence of mercury in seafood, the fish intake of Japanese women has gradually decreased [11]. Therefore, lesser consumption of fish by Japanese women has led to a lower EPA and DHA intake, leading to a higher proportion of LBW infants.

Muthayya et al. examined the association between fish intake and LBW during each trimester of pregnancy and reported that women who did not eat fish during the third trimester were at a substantially higher risk of LBW deliveries [12]. However, a limitation of these studies was that they did not consider supplemental intake. A previous study reported that 600 mg/day of DHA supplementation after 20 weeks of gestation increased birth weight in a cohort with low fish consumption [13]. Therefore, the dietary and supplemental intake of EPA and DHA must be considered. This study examined the association between the intake of EPA and DHA from diet and supplements in the second trimester and the incidence of LBW deliveries in Japanese pregnant women.

## 2. Materials and Methods

### 2.1. Study Design and Participants

This prospective cohort study was a part of the Japan Pregnancy Eating and Activity Cohort (J-PEACH) study [14]. The J-PEACH study is being conducted at four sites in Japan since February 2020: Tokyo, Osaka, Fukuoka, and Yamagata. The J-PEACH study aims to clarify the association of actual lifestyle conditions and factors during pregnancy with the subsequent health status during pregnancy and the postpartum period. The study participants were 1489 pregnant women who were recruited by the J-PEACH study researchers. The inclusion criteria were age ≥ 18 years and the ability to read and write in Japanese. The exclusion criteria for this study were multiple pregnancies and preterm birth of <37 gestational weeks.

### 2.2. Survey Period and Data Collection Methods

This study used data obtained between February 2020 and October 2022 as a part of the J-PEACH study. In the J-PEACH study, data were collected using medical records and the “Questionnaire,” a web-based survey that collected data longitudinally during the second and third trimesters of pregnancy, and at 1, 6, and 12 months postpartum through a web-based survey. The participants were provided a link to access the questionnaire for each period via email. If the participants failed to complete the questionnaire within 2 weeks of receiving it, they were sent a reminder email. Herein, we used data from the second trimester of pregnancy; moreover, data were collected until 11 October 2022.

### 2.3. Dietary Intake

Nutrition data were collected using the brief-type self-administered diet history questionnaire (BDHQ). The BDHQ has an acceptable level of validity in assessing EPA and DHA intake during the second and third trimesters of pregnant Japanese women [15,16]. The information obtained from the BDHQ was used to estimate the dietary intake of 58 foods, beverages, and their energy. An ad hoc computer algorithm based on Japan’s Standard Tables of Food Composition developed for the BDHQ was used to calculate the selected nutrients. Protein, fat, carbohydrate, EPA, and DHA intakes were energy-adjusted using the density method (%/1000 kcal) to reduce interindividual measurement errors. Additionally, as self-administered dietary survey methods generally report errors because of under- and over-reporting, outliers were identified as 2.5% above and below limits.

### 2.4. Supplement Use

The information regarding the previous month’s usage of DHA and/or EPA supplements was obtained from the questionnaire for the second trimester. The participants were asked ad hoc questions such as “Do you use an *n*-3 fatty acid (DHA and/or EPA) supplement?” and “How often do you use it (every day, 5–6 times/week, 3–4 times/week, 1–2 times/week, sometimes, or never)?”

### 2.5. Participant Characteristics

The medical records of the participants were accessed to obtain information regarding maternal age at delivery, prepregnancy body mass index (BMI), parity, gestational weeks, birth weight, and maternal weight gain during pregnancy. Information regarding annual family income, maternal educational level, employment status during pregnancy, prepregnancy history of alcohol consumption and smoking, and frequency of EPA and DHA supplement intake during pregnancy was obtained from the questionnaires.

### 2.6. Statistical Analysis

Descriptive statistics were used to provide background. Dietary intake during the second trimester was classified into the LBW (<2500 g) and normal birth weight (NBW) (≥2500 g and <4000 g) groups, and the Student’s t-test was performed. The analysis was conducted separately for all pregnant women and also for male and female children. The following methods were used to analyze the EPA and DHA intake: First, the DHA and EPA quantities acquired from each food group were calculated and summed. The reason for the sum is that EPA and DHA are types of *n*-3 fatty acids and follow the same metabolic pathway. Next, the participants were divided into high-, medium-, and low-intake groups based on their total EPA and DHA dietary intake. Finally, the supplementary intake of EPA and DHA was considered. The supplement independently purchased by the study participants contained 45–110 mg EPA and 200–406 mg DHA per tablet, with a recommended intake of 2–4 tablets per day. According to the primary observations of the J-PEACH study, maternal EPA and DHA blood levels were noted to be significantly higher when supplements were consumed ≥5 times per week compared with when they were consumed ≤4 times per week. Thus, women who consumed supplements ≥5 times a week were classified as the high-intake group, regardless of their dietary intake. Statistical analysis was performed using the Cochran–Armitage trend test to examine the trend between EPA and DHA intake and birth weight during the second trimester.

Exact tests were performed when the expected frequencies were ≥5; LBW was the dependent variable and total EPA and DHA intake was the independent variable. The analysis was performed separately for all pregnant women and the different sexes of children. The data were analyzed using JMP Pro Ver 16 statistical software (SAS Institute Inc., Cary, NC, USA). The significance level was 5%.

## 3. Results

### 3.1. Participants

**Figure 1:**
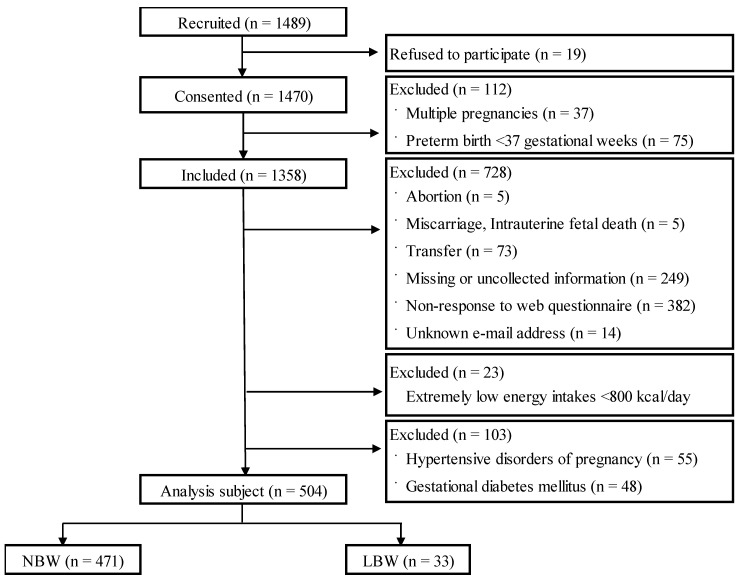
Flow chart of the study. NBW: normal birth weight; LBW: low birth weight.

Among the 1470 women who consented to participate in this study, 1358 were included after excluding multiple pregnancies (*n* = 37) and preterm births (*n* = 75); 728 participants were further excluded because of the following factors: abortion, miscarriage, intrauterine fetal death, transfer, missing or uncollected medical record information, nonresponse to the web questionnaire, and unknown email address (Figure 1). Additionally, 23 participants who were identified with having deficient energy intakes (<800 kcal/day) were excluded. None of the participants reported an extremely high energy intake (≥4000 kcal/day).

Furthermore, the participants with hypertensive disorders of pregnancy (*n* = 55) and gestational diabetes mellitus (*n* = 48) were excluded because they could be confounding factors for LBW. Therefore, a total of 504 participants were included in the analysis: 471 in the NBW group and 33 in the LBW group.

### 3.2. Participant Characteristics

Table 1 shows the characteristics of the participants. The mean ± SD maternal age at delivery was 34.2 ± 4.5 years. The mean prepregnancy BMI was 21.3 ± 3.3 kg/m^2^, weight gain during pregnancy was 9.9 ± 3.9 kg, and the number of gestational weeks was 38.9 ± 1.1 weeks. There were 247 (49.0%) primiparas and 257 (51.0%) multiparas. Among the participants, 237 (47.0%) infants were boys and 267 (53.0%) were girls. The mean birth weight was 3068.6 ± 370.5, 3145.8 ± 370.9, and 3000.1 ± 356.4 g for all the participants, boys, and girls, respectively. Of all the participants, 33 (6.5%) belonged to the LBW category, among which 8 (1.6%) were boys and 25 (5.0%) were girls. In total, 185 participants (36.7%) had an annual family income of JPY > 9 million, and 337 (66.9%) had a university or graduate degree. In addition, 178 participants (35.3%) were unemployed during the second trimester, and 380 (75.4%) reported to have abstained from consuming alcohol or drank less than once a week before pregnancy. The response to smoking during pregnancy was “never” for 449 (89.1%) participants and “smoked during pregnancy” for 2 (0.4%) participants. In total, 44 respondents reported their frequency of taking EPA and DHA supplements during pregnancy as “more than five times a week.”

### 3.3. Intake of Nutrients

Table 2 shows the intake of nutrients in the second trimester of pregnancy. There was no statistically significant difference in the intake between NBW and LBW groups concerning the sex of the infant for all pregnant women and their newborns.

### 3.4. Total EPA and DHA Intake and LBW

Table 3 shows the total EPA and DHA intake during the second trimester of pregnancy and the incidence of LBW infants. The total EPA and DHA intake was classified into three groups: low-intake (EPA + DHA < 172.3 mg), medium-intake (EPA + DHA > 172.3 mg; ≤374.9 mg), and high-intake (≥374.9 mg) groups. Moreover, the mean values for the low-, medium-, and high-intake groups were 111.2 ± 46.2, 263.2 ± 57.0, and 536.3 ± 182.7 mg, respectively.

The proportion of LBW infants was higher in the low-intake group (*p* = 0.04). Similarly, the trend in LBW incidence concerning the sex of the children was also examined. No significant trend was found for either boys or girls (*p* = 0.27 and *p* = 0.35).

## 4. Discussion

### 4.1. Participants

The incidence of LBW in our participants was 6.5%. In Japan, the incidence of LBW, including multiple and preterm births, is 9.4% [1]. Furthermore, the mean BMI was 21.6 ± 4.0. According to the National Health Survey, the mean BMI of Japanese women in their 20 s–40 s ranged from 21.0 to 22.3, indicating that the data on our study’s participants could be generalized to a great extent [17]. Furthermore, 65.4% of participants had college/graduate degrees, and 54.3% had family incomes of JPY ≥ 7 million. Compared with the participants included in the national survey of women of all generations, the participants of the present study had a high social background, which can be attributed to the fact that women are now going to college, and the current proportion of women in the workforce is more than that in the past [18,19].

### 4.2. Transfer of Fatty Acids to the Fetus during Pregnancy

Unsaturated fatty acids, including EPA and DHA, accumulate in maternal body fat during the first trimester. In the second trimester, the accumulated EPA and DHA are transferred through the placenta through the three-fold activation of lipoproteinases in the placenta. In the third trimester, the accumulation of unsaturated fatty acids and progesterone increases simultaneously; therefore, the amount of EPA and DHA transferred through the placenta increases, promoting fetal growth [20]. Additionally, as EPA and DHA are thought to promote vasodilation by increasing blood flow, they may also be responsible for promoting fetal growth by increasing placental blood flow [21]. Therefore, we assume that the prevalence of LBW babies is higher in pregnant women with a lower EPA and DHA intake up to the second trimester of pregnancy. We believe that it is important for the mother to consume and accumulate sufficient amounts of EPA and DHA in the second trimester of pregnancy to enable the transfer of sufficient amounts of EPA and DHA to the fetus in the third trimester of pregnancy.

### 4.3. Nutrients and EPA and DHA Intake during Pregnancy

The total EPA and DHA intake in the second trimester of pregnant women was 296.6 ± 180.5 mg/1000 kcal in the NBW group and 257.6 ± 175.8 mg/1000 kcal in the LBW group. These quantities are considerably lower than those recommended by the Ministry of Health, Labor, and Welfare [22], which are 1600 mg/day for *n*-3 fatty acids, including EPA and DHA. In the U.S., an intake of 500 mg/day is recommended, which is higher than that in Japan. Therefore, it was clear that the intake of the study participants was low. Furthermore, considering the nutrient intake, the average energy intake observed in this study was considerably lower than the estimated requirement (1950–2300 kcal/day) in the second trimester [22]. It was observed that energy intake in the self-administered questionnaires is usually under-reported by ~11% of men and ~15% of women [23,24]; even with this tendency, it is evident that pregnant women do not receive adequate nutrition.

Usually, the second trimester is an important period when many pregnant women can control their eating habits without experiencing nausea and vomiting, which are often present during the first trimester. However, the underlying reason for the results indicating overall undernourishment in this study could probably be attributed to the difficulty faced by women in the second trimester in consuming an adequately nutritious diet because of the ongoing time constraints owing to work or insufficient daily food intake in the first place. Furthermore, currently in Japan, the BMI of the fertile generation in their 20 s and 30 s is declining. Moreover, ~25% of women have a BMI of <18.5 and are considered thin [17]. This situation suggests that women may unnecessarily diet because of a desire to lose weight, and when such women become pregnant, they may eat the same diet as the one they ate before pregnancy. Therefore, it may be necessary to consider re-examining the reference values in this age of increasing desire for thinness.

Even during low intake, to obtain more EPA and DHA, it would be efficacious to increase the consumption of fish, flaxseed oil, and sesame oil. Salmon and sardines, which contain relatively low levels of methylmercury, are specifically recommended. Additionally, changing the quality of the oil used for cooking can optimize the intake of EPA and DHA. In addition, it is advisable to have sufficient dietary and supplementary EPA and DHA intake to avoid deficiencies.

### 4.4. Role of Medical Professionals

Eating behavior and awareness can change during pregnancy. Medical professionals intervene and objectively review pregnant women’s lifestyle habits. In Japan, with an increasing number of LBW infants, the Ministry of Health, Labor, and Welfare issued Dietary Guidelines for Expectant Mothers (revised in 2021) [24] from the perspective of improving the health of the mother and child. However, the LBW infant percentage remains unchanged, and no major changes have been noticed. The Women, Infants, and Children (WIC) nutrition program conducts interviews to assess the health status of mothers and children every few months and has reported improvements in nutritional status in the United States [25,26]. In Japan, it is important to establish a nutrition program based on the Dietary Guidelines for Expectant and Nursing Mothers and create an environment where appropriate nutritional guidance can be individually tailored for each patient by conducting regular nutritional assessments. Midwives work closely with pregnant women during prenatal health examinations. We consider that providing nutritional guidance based on the level of health awareness and lifestyle of each individual and helping them improve their eating habits by the second trimester can play a part in improving their nutritional status.

### 4.5. Limitations and Issues

This study had some limitations. First, this study did not adjust for all potential confounding factors because it focused on looking at trends in LBW with EPA and DHA intake. The dietary patterns of pregnant Japanese women showed that the intake of fish, which are rich in EPA and DHA, was positively correlated with the intake of fruits and vegetables [27]. LBWs have been suggested to be associated with the intake of these foods [28,29,30], which may be a confounding factor. Additionally, 36.7% of study participants had a family income of JPY ≥9 million, a group that is highly likely to purchase dietary supplements; however, as supplements are purchased at private expense, it might be financially difficult for some people to purchase them. As economic disparity is also a confounding factor for LBW, we must conduct future studies considering such various confounding factors. Second, the observed number of women with LBW babies was relatively small. The sample size was insufficient to adjust the confounding factors. Furthermore, the number of participants was smaller when the sex of the infants was also considered an independent factor. Although there were no significant differences in BMIs based on the sex of the infants, it is necessary to include a large population and conduct epigenomic studies because less nutrition during the fetal period may lead to epigenetic changes that may affect the regulation of gene expression in infants.

The strengths of this study are as follows: First, the J-PEACH study was a prospective cohort study conducted in different locations, which enabled us to adjust for regional factors. Second, by considering the dietary and supplemental intakes of EPA and DHA, we could identify the association between low EPA and DHA intakes in the second trimester and LBW.

## 5. Conclusions

Herein, the proportion of LBW was higher in the low-intake group. There was no significant trend in LBW prevalence concerning gender. Low dietary intake and a lack of sufficient EPA and DHA supplementation in pregnant Japanese women until the second trimester were risk factors for LBW incidence. These findings suggest the need for nutritional guidance to ensure adequate intake of nutrients, including EPA and DHA, during the second trimester of pregnancy.

## Figures and Tables

**Table 1:** Participant characteristics.

Parameters		Mean ± SD	*n* (%)
Age at delivery (years)		34.2±4.5	
Prepregnancy BMI (kg/m^2^)		21.3±3.3	
Maternal weight gain during pregnancy (kg)		9.9±3.9	
Number of gestational weeks (week)		38.9±1.1	
Parity	Primipara		247 (49.0)
	Multipara		257 (51.0)
Sex of the children	Boys		237 (47.0)
	Girls		267 (53.0)
Birth weight (g)	All participants	3068.6±370.5	
	Boys	3145.8±370.9	
	Girls	3000.1±356.4	
LBW (g)	All participants	2395.3±418.3	33 (6.5)
	Boys	2412.3±72.4	8 (1.6)
	Girls	2389.9±114.0	25 (5.0)
Family income (JPY per year)			
	<3 million		18 (3.6)
	3–<5 million		105 (20.8)
	5–<7 million		107 (21.2)
	7–<9 million		89 (17.7)
	≥9 million		185 (36.7)
Education	Junior high or high school		46 (9.1)
	Junior/technical college		121 (24.0)
	University/graduate school		337 (66.9)
Working during the second trimester	Yes		326 (64.7)
	No		178 (35.3)
Drink before pregnancy	No drink or <1 time/a week		380 (75.4)
	2–3 times/a week		60 (11.9)
	4–5 times/a week		30 (6.0)
	≥6 times/a week		34 (6.7)
Smoking	Never		449 (89.1)
	Stopped before this pregnancy		43 (8.5)
	Stopped during this pregnancy		10 (2.0)
	Smoked during pregnancy		2 (0.4)
Frequency of EPA and DHA supplement intake during the second trimester			
	≥5/a week		44 (8.7)
	<4/week		16 (3.2)
	No		444 (88.1)

SD: standard deviation; BMI: body mass index; LBW: low birth weight.

**Table 2:** Intake of nutrients in the second trimester.

	All Participants (*n* = 504)			Boys (*n* = 286)			Girls (*n* = 267)		
	NBW (*n* = 471)	LBW (*n* = 33)		NBW (*n* = 229)	LBW (*n* = 8)		NBW (*n* = 242)	LBW (*n* = 25)	
	Mean ± SD		*p*	Mean ± SD		*p*	Mean ± SD		*p*
Energy (kcal)	1545.8 ± 407.4	1516.1 ± 524.7	0.70	1573.6 ± 410.2	1408.4 ± 374.2	0.26	1519.5 ± 403.8	1550.6 ± 566.7	0.73
Protein (g/1000 kcal)	57.5 ± 18.4	55.6 ± 23.6	0.58	57.6 ± 18.4	49.7 ± 17.2	0.23	57.4 ± 18.4	57.5 ± 25.3	0.97
Fat (g/1000 kcal)	50.1 ± 15.1	48.3 ± 17.5	0.52	51.0 ± 15.2	46.1 ± 10.1	0.37	49.3 ± 14.9	49.1 ± 19.4	0.95
Carbohydrate (g/1000 kcal)	212.3 ± 63.1	208.2 ± 65.9	0.72	217.0 ± 65.3	196.1 ± 57.0	0.37	207.9 ± 60.8	212.1 ± 69.1	0.75
EPA + DHA (mg/1000 kcal)	297.2 ± 185.4	240.7 ± 165.7	0.10	289.2 ± 192.9	216.9 ± 69.1	0.29	304.7 ± 178.2	248.3 ± 187.0	0.15
EPA (mg/1000 kcal)	104.9 ± 74.3	83.2 ± 66.52	0.10	101.5 ± 77.4	75.4 ± 29.8	0.34	108.0 ± 71.4	85.6 ± 74.5	0.14
DHA (mg/1000 kcal)	192.2 ± 111.6	157.5 ± 100.0	0.08	187.7 ± 116.0	141.4 ± 40.1	0.26	196.7 ± 107.4	162.7 ± 112.9	0.14

NBW: normal birth weight; LBW: low birth weight < 2500 g; DHA: docosahexaenoic acid; EPA: eicosapentaenoic acid.

**Table 3:** EPA + DHA intake during second trimester and contingency table for low birth weight.

Classification	All Participants (*n* = 504)			Boys (*n* = 237)			Girls (*n* = 267)		
	NBW (*n* = 471)	LBW (*n* = 33)		NBW (*n* = 229)	LBW (*n* = 8)		NBW (*n* = 242)	LBW (*n* = 25)	
EPA + DHA	*n* (%)	*n* (%)	*p*	*n* (%)	*n* (%)	*p*	*n* (%)	*n* (%)	*p*
Low-intake group ^†^	114 (90.5)	12 (9.5)	0.04 *	58 (95.1)	3 (4.9)	0.27	56 (86.2)	9 (13.8)	0.35
Medium-intake group ^†^	236 (93.7)	16 (6.4)		115 (95.8)	5 (4.2)		121 (91.7)	11 (8.3)	
High-intake group ^†^	121 (96.0)	5 (4.0)		56 (100.0)	0 (0.0)		65 (92.9)	5 (7.1)	

Cochran–Armitage trend test. ^†^ Classification of the total EPA and DHA intake: low-intake group: <172.3 mg; medium-intake group: >172.3 mg, ≤374.9 mg; high-intake group: ≥374.9 mg. * *p*: *p*-value < 0.05; NBW: normal birth weight; LBW: low birth weight < 2500 g; DHA: docosahexaenoic acid; EPA: eicosapentaenoic acid.

## Data Availability

The data presented in this study are available upon request from the corresponding author. The data are not publicly accessible.
